# Selections

**Published:** 1881-07

**Authors:** 


### SELECTIONS.

### A CASE OF PERITONITIS.

A CLINICAL LECTURE,

By H. C. Wood, M. D.

This patient was suddenly seized with the most violent purging three days ago. The discharges were free and watery, and so constant that he was forced to remain at stool for two hours. This attack began about mid-day. In the evening the patient suffered from intense abdominal pain. By the following morning this pain had become unbearable. The tenderness below the umbilicus was excessive. There was free vomiting. Now, a great many high authorities contend that there was no such thing as true idiopathic peritonitis. In positive opposition to this view, I hold that I have seen quite a number of idiopathic cases in my own practice. I know no reason why the peritoneum should not share the fate of the other serous membranes. I saw the patient on the morning of the second day, and was quite puzzled in making out my diagnosis. The first questions I put to myself were : Is it a case of idiopathic peritonitis, or is there some other cause for the symptoms? Can they be the result of the passage of a biliary or a renal calculus ? After thoroughly sifting all the possible explanations, I came to the conclusion that it was a case of enteritis, which had extended to the peritoneal covering and brought on peritonitis. The seat of the pain was certainly not that which marks and accompanies the passage of a renal or biliary calculus. The most intense pain was situated low down in the umbilical region. This pain did not run towards the back or penis, as in the case in renal calculus. Moreover, in these diseases, I should not have found such intense local tenderness. There was a possibility of intussusception, which does occasionally occur in adults, but such an explanation seemed improbable : for (1) there was no passage of blood by stool ; (2) there was no suppression of urine ; and (3) there was no local tumor to be found. Could it be an instance of typhlitis? The tenderness was not greater on the right than on the left side, and the locality of the pain was not that of typhlitis. Another strong point against the supposition of the existence of any of the above diseases was that none of them begin with free purging as has been the case here. Free purging is invariably a sign of some bowel complication. I had, some time ago under my care, a woman who had been suddenly seized with the most violent cramping and spasmodic pain in the abdomen. The only thing which gave relief was pressure. Her whole belly was exquisitely tender. All these symptoms had been the result of the attack. I had to deal with a well-marked instance of peritonitis. Now, as regards the treatment I employed. This has been my fifth case of idiopathic peritonitis in private practice. I have seen many cases of peritonitis in puerperal women and in the Almshouse Hospital. In the Almhouse Hospital everybody is utterly broken down in constitution, and so no criterion of an acute disorder can be had there. In all of the five cases above mentioned I have had complete cures. My treatment is nothing new; on the contrary, it is very old. I remember my uncle, Dr. Geo. B. Wood, saying that he never lost a case of peritonitis in an adult, and the reason he gave was that he always bled his patients from the arm until they fainted, and then put one hundred leeches on the abdomen. I am proud to say that I am a thorough believer in the same plan of treatment, antiquated as it may appear. In my first case, that of a woman, as I was not called in until the disease was of many hours' standing, I found bleeding unnecessary, but I did not put on as many leeches as the abdomen would hold. In my second case I also leeched very heavily, it being too late to bleed. In my third case I drew thirty-two fluid ounces by leeches. The patient's cheeks grew deathly pale, her pulse was reduced, and she was left weak and relaxed, but made an excellent recovery. So with the fourth case. In my fifth case the excessive purging rendered the subject scarcely a fit one for venesection. I, therefore, took away only fifteen fluid ounces, thinking that the purging had already weakened the man sufficiently. I have never, you see, had cause to regret having bled my patient copiously. It makes very little difference whether you take the blood from the arm or from the abdomen, provided you draw enough to make a profound impression. You all know that in surgical practice a laige number of local inflammations are subdued by obstructing their supply of blood. The inflammation subsides as soon as the passage of blood is prevented. I do not see why I should not apply this precise principle in cases of peritonitis. For just as a fire goes out when you take away its fuel, so venesection must, and, in my experience, has had a most salutary effect. I have said that it makes but little difference whether you take the blood from the arm or the abdomen. Taking the blood from the abdominal walls should certainly give a more direct effect, though this is not always the case. What is to be done after venesection? I take my stand on the old theory that calomel has power to modify inflammatory action. In cases of iritis the microscope has positively revealed the absorption of the lymph following its use. We know that calomel renders the blood less coagulable and stops the production of fibrin. In inflammation there is a great tendency to the production of fibrin of a low order, which is very likely to coagulate. I am, as you see, a most entire believer in the anti-phlogistic properties of calomel—not, indeed, in inflammation where there is too little fibrin, but in all inflammations where it is in excess. As perotinitis is an exceedingly severe disease, and means death in ninety-nine out of a hundred cases unless they are treated promptly and efficiently, mercury, to do any good, must be taken in decided doses. In my fifth case, referred to above, I gave half a grain of calomel every hour. In this case I am giving one-quarter of a grain every hour. In connection with the calomel, opium is undeniably of great value. We do not know exactly how the opium does good, except that as horrible pain breeds physical exhaustion, so opium puts a stop to the pain and to the exhaustion and restlessness accompanying it. Opium may, therefore, be said (1) to prevent exhaustion, and (2) to cause quiet. I think, indeed, that it does even more by influencing beneficiently the inflammatory action. If you give opium at all in peritonitis give it in large doses. Use enough to keep the patient on the verge of narcotism. Opium, like every other medicine, has no fixed dose, but must be admininistered until its effects are noticed. Never administer opium in the form of hard pills, for the pills collect, unsoftened and unactive, in the alimentary canal, until the whole mass is suddenly dissolved and thrown into circulation. I, therefore, advise its use in the liquid form, and in repeated small doses. The ability to stand large doses of opium in peritonitis is wonderful. In one of my cases seventy-five grains of solid opium were taken daily for five days, and the patient made an excellent recovery. In another instance twenty drops of deodorized laudanum were repeatedly injected into the rectum for a long period of time. As the disease wears out, the ability of the system to stand large doses subsides, so that the quantity must be gradually diminished. Dr. Binz, in his latest work on therapeutics, makes the point-blank assertion that no one has satisfactorily proven thus far that counter-irritants do good in disease. In reply to this statement I desire to advance the argument that no one who has had a severe stomachache will say that he does not believe in counter-irritation. Therefore, by all means, use blisters in peritonitis, but never use blisters in the early stages of the disease. The first thing to be done when the leeches have been removed is to apply poultices, whether they are hot or cold makes little difference. Where there is a very marked tendency to feverishness it is perhaps better to apply cold poultices. If the abdomen is too tender to bear the weight of the ice-bag apply light flannel cloths wrung out of ice-water. In this case I have been using light cloths wrung out of hot water. A warm-water dressing may act merely as a local derivative to the skin ; but I think it probable that some of the warm-water oozes through the intervening tissues into the abdomen, and so acts directly as a soothing agent upon the inflamed peritoneum. Do not, however, understand me as making a positive assertion with regard to this point. That warm and local applications do most certainly affect the neighboring and adjacent tissues to a considerable depth I have amply proven by numerous experiments on animals. The application of it to the head of a cat, for example, will affect the base of the brain very perceptibly. So, too, as regards external application of either moist or dry heat. After the abdomen has been thoroughly poulticed for two or three days blisters may be used, provided the temperature of the body has not remained high—that is, a blister may be applied at the end of ten days if the temperature has fallen in the meanwhile. Do not put on a small blister. I was talking with my uncle, Dr. George B. Wood, the other evening, about this very case, and he said that if he were in my place he would order a blister ten by ten (inches). I have ordered a blister eight by ten. In one of my first cases pus had formed in the abdomen, and I blistered so freely that the woman got tired of me and called in the services of an old Indian woman, who told her that her liver strings were loose. Her treatment, like mine, consisted in persistent blistering. As soon as one spot of new skin showed itself the old woman would clap on another blister. After some more experience of the Indian doctress, the patient determined to have me back again. I saw that though the herb-woman's diagnosis was faulty her therapeutics were good, and by sticking to the blisters for some time longer I succeeded in curing my patient entirely. How does quinia act in peritonitis? you will ask. There is a very prevalent belief that large doses of quinia are of service in this disease. I have seen most remarkable results from its use in puerperal peritonitis. There it does good by its action on the septic material, expelling it by causing contraction of the womb. Quinia might be of advantage in some cases of idopathic peritonitis. Generally the stomach is not strong enough to bear it, such has been the case here. As regards food the less you give a patient in the first few days of an attack the better. When you do begin to feed remember that you are feeding a patient whose abdominal contents are all glued together with adhesive lymph ; and also remember that if these agglutinations are torn apart there is great danger of hemorrhage or collapse. The food which should be given after the first acuteness of the attack has passed off must be that which leaves the least residuum of undigested matters, and, therefore, causes the least amount of peristaltic action on the part of the intestines. Milk in repeated small doses is the best article of food possible. After the end of a few days you may give some solid article. If there are symptoms of exhaustion late in the course of the attack give beef-tea as a stimulant. Alcohol is not only powerless but even dangerous in the early stage of the disease. A few doses of brandy in the first days of an attack of peritonitis may produce death. This patient took a couple of tablespoonfuls of brandy at the beginning to relieve the griping, and he says it burnt him like fire. Alcohol, of course, only adds fuel to the fire. A very important question arises during convalescence as to how the bowels shall be open. Never think of using a purgative or an enema. These bring violently into play all the muscles of the abdomen. Very often there will be a spontaneous movement on the fifth or sixth day without any medicine at all. If there is not such an opening give at the end of ten days a small dose of castor-oil. If there is retention of urine the water must of course be drawn off by means of the catheter. In using calomel in the early stages, my advice to you is to push its use to the production of slight ptyalism and soreness of the gums. If you follow out the plan of treatment which I have sketched for you in all its details I think you will treat the disease very successfully. In concluding, let me point out how far this treatment is applicable to cases of puerperal peritonitis. I am convinced that there is a class of cases in which it is desirable. Where there is a wave of puerperal peritonitis due to adynamia flowing over a section of country this treatment will be of no avail, for here the disease is only an expression of a blood disorder. Nor is this treatment well borne where the disease is plainly septic in its origin. There is, however, a third class in which the disease has been brought on by bruising of the parts during the process of labor. In this class the treatment for idiopathic peritonitis is well borne. These are the cases resulting from an extension of non-septic metritis. Here, of course, free venesection does great good. In these cases there is always decided febrile reaction, but absence of characteristic puerperal typhoid and adynamic expression of face. In such cases you should always superadd the use of quinia, so as to provoke uterine contraction and so prevent septicæmia. You must have great care during convalescence from peritonitis to prevent a relapse. Do not think of allowing any violent or gymnastic exercise for a long time afterwards. Fibrinous bands have been formed in the abdomen and may have become the seat of blood-vessels. If these are torn you may have either acute hemorrhage or a very serious relapse.—*Medical Gazette.*

### ON FILTH AND SEMI-FILTH DISEASES.

BY JOHN C. PETERS, M. D., NEW YORK.

I have coined the name semi-filth diseases, in order to cover a large class of disorders which may arise from other causes, but in which filth is only too often a large factor.

The great sources of filth in large cities are dirty streets and gutters ; the large quantity of filth which is washed down into the receiving basins and sewers with every rain-storm ; and the fouling of dock-grounds and water by the contents of the sewers. There is every reason to believe that more filth in the shape of garbage and slops gets into the sewers from filthy streets and gutters than from water-closets and kitchen sinks, and that a very large proportion of sewer gas is thus caused by filthy streets and gutters. Next in order is the vile odor from out-door privies, many of which are without any ventilation, having neither windows nor chimneys. The health authorities are only too often remiss in their attention to these nuisances. London has one water-closet for every five inhabitants. It is not at all uncommon to find water-closets, even in otherwise good houses, without windows or other means of ventilation, except by the door only, which must, of course, be kept closed when in use. Dirty cellars and foul air streaming up from the gutters into the air-boxes of almost all houses, are other sources of household sickness. The stables of great cities are only too often in a filthy condition, and in this they are very rarely inspected by the health authorities. The smokes and smells from gas and ammonia works, from offal-rendering establishments, and the making of fertilizers, are pregnant sources of discomfort and disease. It, is, perhaps, not commonly known that the fertilizer-making establishments, which cause so many mal-odors in this city, use ground bones, blood, all the offal and scrap, and the contents of the bowels of slaughtered cattle. These are first boiled, then dried, and much foul gas from them is not consumed, but escapes from the chimneys, and are generally attributed to Hunter's Point.

Foul air must first attack the throat, air-passages, and lungs. Diphtheria has often been traced to sewer-gas, but much more commonly arises from foul streets, gutters, drains, receiving-basins, etc.

Although it and membranous croup are often apparently excited by cold, yet they more commonly arise from cold taken in foul air, and must be regarded as partial or semi-filth diseases. In 1880, 72 deaths occurred from diphtheria in January ; 77 in February ; 65 in March ; 81 in April ; 76 in May ; 61 in June ; 89 in July ; 97 in August ; 125 in September ; 199 in October ; 234 in November ; 214 in December. So that they have some other factor besides cold, and that probably is filth.

*Consumption* is generally regarded as a chronic catarrhal pneumonia, most often caused by cold and insufficient clothing ; but in 1880, there were 402 deaths in January ; 375 in February ; 412 in March ; 394 in April ; 365 in May ; 351 in June ; 385 in July ; 380 in August; 376 in September; 408 in October; 339 in November; 450 in December. The deaths are so evenly distributed through every month in the year that there must be some other cause than exposure to cold, and that cause is probably the inhalation of foul air. Bowditch gained great credit for apparently proving that consumption arose from moist ground; but foul moist ground is probably a greater factor, and the inhalation of foul dust and dirt is not far behind it in deleterious effects.

Pneumonia, also, is generally attributed to exposure to cold and wet; but in 1880, 261 deaths occurred in January; 248 in February; 266 in March; 375 in April, and 340 in May. So that it also has another factor than mere cold, and that doubtless is the inhalation of foul air, It is true that only 163 deaths occurred in June; 127 in July; 108 in August; 134 in September; but in those months the city is largely depopulated; 205 deaths are recorded for October; 266 for November; and 349 for December.

From bronchitis, 106 deaths are recorded in January; 122 in February; 140 in March; 138 in April; 131 in May, and 102 in June. So that bronchitis has other causes than taking cold and foul air; bronchitis and pneumonia are well-known diseases.

Typhoid fever is generally accepted as a filth disease. Typhus fever arises from the over-crowding of filthy people, and is at least a semi-filth disease. Cholera is a well-known filth disease; and cholera infantum arises as much from foul, hot air as it does from spoiled food or mistakes in diet, and is at the very least a semi-filth disease. Yellow fever is now generally admitted to be a filth disease, prevalent only in dirty cities and places; and all malarious diseases are necessarily foul-air or filth diseases.

Civic malarious diseases, arising from the combined influence of foul ground and subsoil, foul streets, gutters, drains, receiving basins, cellars, back yards and privies, and other baneful influences, are certainly filth diseases. Pure, fresh air and free ventilation are necessary in the treatment of all diseases, and foul air increases the malignancy and mortality of all infections and contagious diseases, including smallpox, measles, scarlet fever, diphtheria, whooping-cough, typhus and typhoid fever, and many others.

These positions are so true as to be regarded as axiomatic by all except exceedingly old-fashioned medical men or obstinate officials.

Let the Street-cleaning Department give us clean streets, gutters and receiving-basins ; and the Board of Health give us wholesome outdoor privies, clean stables, control noxious trades far better than it does, and abate the loathsome smokes and smells which abound here, and then the death and sick rate will rapidly fall. The unhealthy condition of the city may be very equally charged upon the negligence of both these departments.

Puerperal diseases are attributed to other causes besides taking cold, yet we find 40 deaths recorded in January, 1880 ; 32 in February ; 37 in March ; 49 in April ; 42 in May ; 24 in July ; 24 in August ; 37 in September ; 24 in October ; 20 in November, and 45 in December.

The acclimatization to filth is a curious problem. Some people become accustomed to it, and thrive as well upon it as others do on tobacco and whiskey ; but 3,469 children died of diarrhœal disease in 1880 ; and no less than 14,690 children died under five years of age. The great majority of these lived not only in poverty, but in filth. The deaths of children in filthy cities reaches enormous proportions, and those who survive may thrive like rose-bushes and potato plants in manure heaps.—*Medical Record.*

### MEDICATED GELATINE IN THE TREATMENT OF NASAL CATARRH.

Read before the Philadelphia Laryngological Society

BY DR. ISAAC BARTON.
Chief of the Throat Department, Jefferson College Hospital.

By this I mean the use of the so-called nasal bougie, which consists of a piece of moulded gelatine, about two or three inches in length, and having a piece of string running through irs entire length, and having a free quantity for the purpose of security. For use, the bougie is to be slightly coated with sweet oil, and gradually inserted by rotation, into either side of the anterior nares. It may then be secured by tying the free quantity of string around the ear, or fastening the string to the cheek by a small piece of adhesive plaster. The head of the patient is then to be inclined backward, and as the gelatine dissolves and flows from the posterior nares into the throat the patient may expectorate at pleasure.

These bougies dissolve in from ten to fifteen minutes.

The following are the medications I have used, which were made for me by Mr. C. L. Mitchell:

| | | |
|---|---|---|
| No. 1. | Liq. iod. comp. | *m*v. |
| 2. | " " " | *m*x. |
| 3. | Morph. sulph. | gr. ¼. |
| 4. | Zinci sulph. | gr. ij. |
| 5. | " " | gr. ij. |
| | Acid carbolic | gr. ss. |
| 6. | Ext. krameria, fld. | gr. viij. |
| 7. | Zinci sulph. | gr. ij. |
| | Acid carbolic | gr. ss. |
| | Ext. hydrastis, fld. | gr. v. |
| 8. | Iodoform | gr. ij. |
| 9. | Liq. iod. comp. | *m* x. |
| | Acid carbolic | gr. j. |
| 10. | Salicylic acid | gr. j. |

From all these medications I have received the best results from Nos. seven, nine and ten.

J. K., aged 23 years, applied to me complaining for nearly eight months of discharge from the back portion of the nostrils into the throat, which discharge was of a thin, mucilaginous character; his general health good; no specific history; sometimes suffered with slight headache. An examination revealed the nares to be moderately filled with mucus. I washed out the nares with Thudichum's nasal douche, after which I inserted two bougies (one into each side of the anterior nares) medicated with zinci sulph., gr. ij., carbolic acid, gr. ss, and hydrastis, fld., *m*v. These dissolved in fifteen minutes. I made this man come to me three times a week, and inserted these bougies after his using the douche, on the same day. At the end of three weeks there was no discharge and had been no headache.

I have used these bougies with some good results in cases of ulceration of the nasal septum.

Clara G., aged 26 years, applied to me, complaining of great pain on left side of nose, which she had four or five days. On examination I found a somewhat superficial ulcer, about the size of three-cent piece, on the left side of the septum. This I carefully cleaned with absorbent cotton, inserted a bougie containing ¼ gr. morph.

sulph. which dissolved in twenty minutes. I used one of the same kind on the next day, after which she had no pain. On the third day I used one medicated with two grains of iodoform (which takes half an hour to dissolve), and continued the use of the same daily, for a week, after which time the ulcer had healed very nicely, the patient having no pain or difficulty in breathing through the nostrils. —*Med. & Surg. Reporter*, April.

Sir William Jenner, M. D., K. C. B., is the new president of the Royal College of Physicians of London.

### THE FATE OF THE SPRAY.

"It is now ten months since the use of the spray has been abandoned in Billroth's clinic. The experiment has proved in the highest degree satisfactory. Operations are even more successful than when performed under spray, and a troublesome piece of apparatus is dispensed with. Since September Billroth has done laparotomy eleven times. Two of the operations were hysterotomies, one per vaginam, the other through the abdominal walls. The remaining nine were ovariotomies. These operations were all performed in the General Hospital, without spray or irrigation, and recovery in each case was perfect."—Vienna Correspondent, *Boston Med. & Surg. Jour.*

### NEW ORLEANS TO BE SEWERED.

The New Orleans *Times* announces the vote of the city council adopting the Waring system by a vote of five to two, and adds: "The city is to be sewered. We want all the world to know it. The city is to be drained. We want all the world to know it. The city is to be clean, sweet, fragrant with rose-blossom and orange-blossom, with jasmine and lily-bell, and we want all the world to know it. The city is to be the healthiest on the continent, and we want all the world to know it."

### SUGGESTIONS IN TOOTHACHE.

At the last meeting of the Odontological Society of Great Britain,

Mr. Stockton read a paper on "The Value of Certain Remedies in the Conssitutional Treatment of Inflammatory Conditions of the Vascular Tooth-Structure, and of Neuralgia arising therefrom." The remedies to which Mr. Stockton specially directed attention were chloride of ammonium, sulphide of calcium, and gelseminum. He had selected them because their action was not so generally known as that of many other agents. He gave a full description of the therapeutic effects of these drugs, indicating the class of cases in which each would be likely to be most serviceable. His conclusion was that, in simple neuralgia of the fifth pair, gelseminum, either with or without aconite, would effect a cure, or at least, afford considerable relief. If the pain was due to congestion or inflammation of the pulp or periosteum, he would prescribe also chloride of ammonium. While in chronic periostitis with suppuration, sulphide of calcium gavs results which were in the highest degree satisfactory, cutting short the attacks in the most remarkable manner. He was of opinion that dental surgeons do not generally give sufficient attention to the constitutional treatment of the cases under their care.

### IMPORT OF THE SWEATING OF CONSUMPTIVES.

Dr. Rousselot, of Saint-Die, discusses, in the *Revue Méd. de l'Est* some of the peculiarities of phthisical sweating, the variable period of the appearance of this symptom, and the point whether the sweating of phthisical persons is to be considered an evil symptom and one which is to be combated. M. Rousselot believes that in a certain number of cases there is a correlation between the sweating and the fever. He remarks, in the first instance, that nothing is more variable than the period of appearance of the sweating in the course of pulmonary phthisis. There is an active tubercular evolution and a torpid evolution in some sort passive. In the second case pulmonary lesion has no influence on the organism. It has not an effective evolution, and it may last for some time without producing fever, and, in consequence, without the procession of symptoms which are ordinarily observed with fever, and particularly in nocturnal sweating. When, on the contrary, there are from the outset an active evolution, nocturnal fever, and disorded condition of all these symptoms, then in general a hasty appearance of nocturnal sweating may be observed.

In this case the thermometer will render great service in enabling us to study the degrees of morbid combustion. The sweating, which is then very often extremely abundant, allows the elimination of a great quantity of the products of morbid combustion. It may then be admitted, according to M. Rousselot, that the sweating affords a derivation favorable to the fever, and does to some extent moderate that symptom. If, then, in certain tubercular persons nocturnal sweating appears, as it were, at the outset of the affection, it is because these individuals have a tuberculous evolution of an active form, and one which tends to fluxion and precocious fever. In others, on the contrary, the evolution effects a silent, indolent, torpid form, without any recoil on the organism ; or more strictly, there are tubercles in the lung, but no tuberculous evolution, and the subject is not phthisical. —*The Med. Gazette.*

### "TINCTURA ANTACRIDA."

This is the name of a secret prescription employed by a Wheeling physician for secondary syphilis, and can only be filled by one druggist in the city. On that account it has gained some notoriety hereabouts, and sometimes unenviable comment. The following is the original recipe and directions :

℞. Gum guaiac, one ounce ; Canada balsam, one ounce ; ol. sassafras, two drachms ; merc. corrosiv. sublimat., one scruple ; rect. spts. vini. (alcohol), eight ounces. Dissolve the guaiac and balsam in one-half the spirit, and the corrosive sublimate in the other. Let the guaiac and balsam digest for several days ; then pour off the clean liquor, mix with the sublimate and add the oil.

"*Dose.*—Ten or twenty drops, night and morning, in a glass of wine or water, *pro re nata.*"

The prescription is a very old one. More than a half a century ago the elder Dr. Fenner got it out of an old English work, then extinct, written by a Dr. Falk, of London. It was recommended as an excellent emmenagogue, more especially "in that painful form of menstruation called dysmenorrhea, and that it was remarkable for almost invariably causing fruitfulness in the case of young married women."

The same prescription may be found in Ellis' Medical Formulary, but it is there placed under the head of alteratives.

Dr. Farmer says : " I usually direct the patient to begin a day or two before the expected period, and take twenty-five drops in an infusion of sage or sweetened water, night and morning, until the discharge is freely established ; then cease till the next period. In obstinate and severe cases, the medicine should be commenced a week or ten days before the period ; and if the pain appears, the medicine should be taken every four or six hours until relieved. * * * I have given it to my brother practitioners wherever I have lived, and they have all pronounced it the best remedy they ever used for this complaint."—*Medical Recorder.*

### METHOD OF EXCITING CARDIAC CONTRACTIONS IN CASE OF SUSPENSION OF THE HEART'S ACTION.

When I was a child, relates Dr. J. C. Reid, I remember that my father received a summons from near by to visit a young woman who was in a fit. My father was absent, but soon returned, when a messenger announced to him that it was too late, that the patient was dead. Nevertheless, he immediately repaired to the patient's abode and ordered some warm water, which he caused to flow from a height on the præcordiel region. Little by little the movements of the heart returned, and the patient was restored.

This recollection of his boyhood days recently caused the author to make use of the same remedy in the case of an old man who appeared to have succumbed in a fit, and in whom the cardiac pulsations had ceased, and the treatment restored the old man to life.—(*Brit. Med. Journal*)—*Bulletin General de Therapeutique.*

### TREATMENT OF HYDROCELE IN CHILDREN.

In the *Courier Medical* Dr. Ange gives a method of treatment which he has applied a great many times to children aged from two to eight years, and *never* had recourse to tapping to effect a cure. His method is sufficient to cause absorption of the fluid. It consists in applying daily, or every other day, Elastic Collodion to the sac, or along the cord if it be a hydrocele of the cord. The same means tried upon a young man aged eighteen, and upon several adults, failed in each case. From this it appears to be only applicable to children. —*Clinical Review.*

### COCO-NUT AS A REMEDY FOR TAPEWORM.

In the Antilles the coco-nut is a popular native remedy for tapeworm, and its utility has been proved by Dr. Martiali, chief of the Medical Service at Senegal. A nut of the coco (Coco crucifera) weighs about five ounces, and the whole of this is taken, scraped, and three hours afterwards a dose of castor oil is administered. The worm comes away five or six hours after the dose of coco-nut. In nine cases in which it was given the remedy was successful.—*Lancet.*

### INTRODUCTION OF WHEAT INTO AMERICA.

Prior to the discovery of this continent by Columbus, there was no cereal in America approaching in nature the wheat plant. It was not until 1530 that wheat found its way into Mexico, and then only by chance. A slave of Cortez found a few grains of wheat in a parcel of rice and showed them to his master, who ordered them to be planted. The result showed that wheat would thrive on Mexican soil, and to-day one of the finest wheat valleys in the world is near the Mexican capital. From Mexico the cereal found its way to Peru. Maria D'Escobar, wife of Don Diego de Chauves, carried a few grains to Lima which were planted, the entire product used for seed for several successive crops. At Quito, in Equador, a monk of the order of St. Francis, by the name of Fray Iodosi Rixi, introduced the new cereal; and it is said that the jar which contained the seeds planted is still preserved by the monks of Quito. Wheat was introduced into the present limits of the United States contemporaneously with the settlement of our country by the English and Dutch.—*Phrenological Journal.*

Connecticut has adopted a law to prevent irregular medical practice, which enacts that any transient person not an inhabitant of the State, who shall treat any disease or injury shall be fined $25 a day for each that he shall exercise his profession without a license. The license may be obtained from the selectment of towns, or the chief officer of police of cities, on payment to the town or city of $20 per day for each day for which the license is granted.—*Sanitary Engineer*.

### ABOUT RICHMOND CROWNS.

BY J. A. ROBINSON, D. D. S., JACKSON, MICH.

Take platinum plate, rolled to 30 or 32 in thickness, and flow scraps of gold foil on one side of it, if you wish to give it a look of gold, and make the hoop of your crown of that, instead of gold. Now why? Because the platinum is stronger, and more easily adapted to a perfect fit round the root, and is not liable to melt in soldering, and will stand any amount of heat without changing form, Finish in the usual way, and you have a stronger crown than one of gold plate, and one that is less expensive.

To make amalgam crowns, not after the plan suggested by Dr. Richmond, take a No. 00 file, and take the *temper* out of it so that it will bend easily round the neck of the tooth, and form a crib of the size and length wanted; place it round the neck of the tooth, and under the free margin of the gum; make it the right height, as you wish it to impinge on the grinding surface of the antagonizing tooth; spring it on to the root, and fill with amalgam, and let it remain for three or four days, or until the whole mass of amalgam is thoroughly hard. If necessary, put in steel screws, the size needed to fill the nerve cavity, for strength; and you will have an "amalgam crown," when finished, that will be comfortably worn, and will do good service. Now, lest some of your readers should suspect this method was a sanction of the "New Departure," I wish to state that I do not think it as good as the Richmond plan, or that, in suggesting this way of making a crown, I am advocating the common use of amalgam, in dental practice; but it is far better than tooth extraction, when the roots are reasonably strong, and fit to have placed upon them any appliance to make them more useful.

To make gold solder, fit to solder gold for Richmond crowns, take equal parts, by weight, of copper, silver, and zinc; melt the silver and copper in a crucible, then add the zinc, in small pieces; and when the blue gas is blown off, caused by adding the zinc, pour off into an ingot and call it *alloy*. To make gold solder from this alloy, take three parts of gold, and one part alloy, and melt together, and it will make a solder that will not change color in the mouth. Neither will it burn when used in soldering and will flow freely on gold plate fifteen carats fine.—*Ohio State Journal of Dental Science.*

### A REMARKABLE MOTHER'S MARK.

A distressing but very interesting case has been reported by one of the surgeons to the Pennsylvania Hospital. A woman thirty years of age, and in the last month of pregnancy, was severely burnt by her clothing catching fire from a kitchen grate. The child was born dead, prematurely, and the mother died two days later from the effects of the burn. The point of interest was that the body of the child was apparently blistered and burnt in extent and in places almost exactly corresponding to the injuries received by the mother. This was of course of the nature of a mother's mark, but how and why the child became thus injured correspondingly with its mother, is a problem yet remaining to be positively explained.

### THE LONG DESIRED RECOGNITION.

During all these years it has been almost, or quite self evident that dentistry is a part of medical science. In its essential nature it could not be otherwise. Denistry requires a knowledge of anatomy, physiology, pathology — but these are all medical sciences. Chemistry is a science common to all the professions and occupations in life, and it belongs alike, therefore, to medicine and dentistry. The dentist needs to understand the fundamental principles of civil engineering ; but so does the general surgeon. The one cannot properly extract teeth, nor the other reduce dislocations without such knowledge. There is no science collateral to general surgery that is not collateral to dentistry.

Many dentists, with commendable industry and most admirable energy have been, for years, in search of recognition from the medical profession proper—such recognition as is now, but has not always been, given to surgery ; and we are glad to say their research has been rewarded with the most emphatic success. And success is the most successful thing in the world.

At the late meeting of the American Medical Association, in Richmond, Virginia, the renowned surgeon, Prof. Gross, moved that the rules be suspended that he might take the necessary steps to organize a section in Dentistry. The motion carried. Through a private letter from a highly distinguished dentist who was present, we learn that some preferred, as the name of the section, the term "Dentology," others "Dental and Oral Surgery ;" but Dr. Gross adhered to the term DENTISTRY, and Dentistry it is. The objection to this term on the part of some, is that it recognizes mechanical as well as operative dentistry. But why should it not ? Dentistry, as a profession, sprang into existence, recognizing the joint study and joint practice of these two departments, if they be two, which is doubtful ; and there never was, and never would have been a dental *profession*, had they not been cultivated in unison ; for their separate consideration had failed, from the dawn of science till the forenoon of the present century.

A young couple of ardent lovers are properly mated and married ; but sometimes the surrounding circumstances develop the mental forces of the one, leaving the other *statu quo.* Now, we care not how much the man may be developed and fitted to shine in the higher walks of life, we have feelings only of contempt for him if he tries to show off where he cannot take with him the wife of his youth.

It has happened in dentistry that, in the last twenty-five years, more scientific thought has been bestowed on operative than on mechanical dentistry ; and operative is seeking a divorce for want of compatibility, or because it is supposed that mechanical dentistry cannot shine in the society of physicians and surgeons. Shame on the man that does not see to it that his wife's social status keeps pace with his own.

But Dr. Gross was the very man to make the motion. His department taken in as surgery—not as surgeology—with its splints, bandages, rollers and other traps, just as dentistry, and not dentology, is taken in with its plates, plaster, and appliances. And, after all, making a mortise in a piece of ivory, and manufacturing a block of gold to fit it, is more mechanical, less physiological, and less scientific than even the selection of teeth for Mr. and Mrs. Jones, to say nothing of their adaptation, which is to restore to the Joneses their natural expressions, or to make them seem as strangers to each other the remainder of their lives. The argument for divorcing the supposed different departments of dentistry has been assumed impossibility of medical recognition for the mechanical branch, if such there be, especially as it was claimed that all the cheap-John quackery pertained to that department, forgetting that in the nostrils of the true medical man the amalgam nuisances and amalgam quacks are a stench surpassing the other as far as the poodle is surpassed by the pole-cat.

Now that the American Medical Association has a section on DENTISTRY, thus recognizing us, let us recognize ourselves as dentists.—*Ohio State Journal of Dental Science.*

### DENTISTS' HEADACHES.

WORKING at the dental chair, as is well known, taxes the eyes very severely. Unfortunately, it often happens that both eyes can not be fixed upon the working point. This, and the fatigue from standing in constrained positions, often bring on a form of headache not peculiar to dentists, but perhaps more common to dentistry than to any other handicraft or occupation. * * * * The pain is often very acute, totally unfitting the victim for professional duty. Even temporary relief is to be hailed as a blessing in such cases. For this we know of nothing better than Trosseau's Formula * * * * * as follows:

℞
Potass. Cyanide . . . . . . . . . . . . grs. iv
Aquæ Lauro-cerasi . . . . . . . . . . ℥ iv
Ft. solut. . . . . . . . . . . . . . . . m
S. Poison. Moisten a compress and apply to seat of pain.
* * * * *Ohio State Journal of Dental Science.*

A YANKEE visiting Colorado says the air is so dry there that everything dries up and nothing rots.

